# Pathway analysis of the impact of health literacy, social support and self-efficacy on self-management behaviors in pregnant women with gestational diabetes mellitus

**DOI:** 10.3389/fpubh.2023.1188072

**Published:** 2023-11-10

**Authors:** Fangmei Tang, Xiaoying Zhong, Sixu Liu, Xiujing Guo, Dehua Li

**Affiliations:** ^1^Department of Nursing, West China Second University Hospital, Sichuan University/West China School of Nursing, Sichuan University, Chengdu, China; ^2^Key Laboratory of Birth Defects and Related Diseases of Women and Children (Sichuan University), Ministry of Education, Chengdu, China; ^3^Department of Nursing, Zigong Fourth People’s Hospital, Zigong, China; ^4^Department of Nursing, School of Medicine, Mianyang Central Hospital, University of Electronic Science and Technology of China, Mianyang, China; ^5^Nursing Department, YAAN People’s Hospital, Yaan, China; ^6^Office of Operations Management and Evaluation, West China Second University Hospital, Sichuan University, Chengdu, China

**Keywords:** gestational diabetes mellitus, health literacy, social support, self-efficacy, pathway analysis, structural equation model

## Abstract

**Objective:**

The purpose of this study was to investigate the pathways by which health literacy (HL), social support, and self-efficacy influence self-management behaviors of pregnant women with Gestational diabetes mellitus (GDM) and the interrelationships between the variables.

**Methods:**

A total of 565 pregnant women with GDM was recruited. The Demographic Characteristics Form, Health Literacy Scale, Perceived Social Support Scale, General Self-efficacy Scale and GDM Self-management Behavior Scale were used for data collection. Descriptive statistics, zero-ordered correlation analysis, and multiple linear regression analysis were performed on the variables; Structural Equation Model (SEM) were constructed for pathway analysis.

**Results:**

A positive correlation was found between health literacy, social support, self-efficacy, and self-management behaviors among pregnant women with GDM after adjusting for age, education level, income level, work status, parity, and family history of diabetes (*r* ranging from 0.203 to 0.533). A further multiple linear regression analysis showed that functional HL, communicative HL, critical HL, social support, and self-efficacy were all independent influences on self-management behaviors and accounted for 36.3% of the variance. Communicative HL and critical HL explained the strongest self-management behaviors (β = 0.316 and 0.255, respectively, *p* < 0.001). The SEM model was suitable for χ^2^/DF = 2.860, RMSEA = 0.060, IFI = 0.953, TLI = 0.943, and CFI = 0.952. The results showed direct positive effects of health literacy on self-management behaviors and self-efficacy, direct positive effects of social support on health literacy and self-efficacy. Social support and self-efficacy have had no significant direct impact on self-management behaviors, but social support may indirectly influence self-management behaviors through the health literacy mediation role.

**Conclusion:**

Healthcare providers should pay attention to the positive impacts of health literacy and social support on self-management behaviors of pregnant women with GDM. Improving the health literacy level of pregnant women with GDM should be the key point of intervention in practice, and the social support system should be fully mobilized to enhance emotional support and life support to promote the improvement of self-management behaviors.

## 1. Introduction

Gestational Diabetes Mellitus (GDM) is one of the most frequent pregnancy complications experienced by women during pregnancy. Its high prevalence and morbidity burden make it a global public health issue (1), and the prevalence of GDM in mainland China is 14.8% (2). GDM increases the risk of perinatal complications such as preeclampsia, premature delivery, cesarean delivery, neonatal hypoglycemia, the future risk of type 2 diabetes and cardiovascular disease (3). moreover, the experience of GDM may increase maternal psychological burden and emotional harm, leading to mental health problems such as anxiety and depression (4). Dietary and nutritional therapy, exercise management, glycemic monitoring and control, fetal monitoring, and postpartum monitoring are key components of GDM management, which are strongly dependent on the patient’s capacity for self-management (5–7). Studies have shown that strict dietary and proper exercise can help patients achieve better glycemic control (8, 9), reduce the incidence of adverse pregnancy outcomes among pregnant women with GDM (10), improv patient’s motivation for treatment, and alleviate patient’s anxiety (11). The current status of the self-management of women with GDM in China is unsatisfactory, with only approximately 30% having good self-management behaviors in some areas (12).

Health literacy (HL) is people’s “motivation, knowledge, and ability to acquire, understand, evaluate, and apply health information in order to make judgments and decisions about health care, disease prevention, and health promotion in daily life to maintain or improve quality of life over the life course” (13). In many countries, improving citizens’ HL is considered one of the goals of strategic plans aimed at improving national health (14), including China (15, 16). There is a close relationship between health literacy and health behaviors, which is a precondition and guarantee for the achievement of healthy behaviors. Patients with low levels of HL were found to have inadequate self-management skills, an increased likelihood of adverse health outcomes (17), and affect the level of glycemic control in diabetic patients (18–20).

Social support and self-efficacy are vital psychosocial concepts related to the health and well-beings of populations closely. Social support always refers to the instrumental, emotional, or informational social resources or help which perceived or received by individuals, is a multi-dimensional concept (21). Individuals often need additional social support resources to cope with illness or other challenging events (22). Studies suggested that low social support is significantly associated with the risk of mental health problems such as depression, anxiety and self-harm during pregnancy (23). Social support also influences pregnant women’s self-management behaviors, a systematic review of qualitative researches showed that lake of social support was one of the barriers to self-management among pregnant women with GDM (24). Self-efficacy is the confidence in the ability to achieve behavioral goals in specified domains, an individual’s belief that he or she can be successful (25), it is a subjective feeling like “I can do it.” Self-efficacy can positively influence patients’ self-management skills and behaviors. In the studies of adults with type 2 diabetes and pregnant women with hyperglycemia, researchers found that higher levels of self-efficacy were associated with better self-management behaviors (26, 27).

There are interconnections among HL, social support and self-efficacy, these variables influence patients’ self-management behaviors. Some studies have explored the mechanisms of factors influence patients’ self-management behaviors. A study revealed that social support and HL were significant predictors of self-management behaviors (28). Another study showed that HL and self-efficacy played a partial mediating role in the process of social support influencing self-management (29). HL has also been found to influence glycemic control and self-management behaviors in patients with type 2 diabetes by direct effects as well as indirect effects mediated by self-efficacy (30–33).

The mechanisms of the influence of HL, social support and self-efficacy on self-management behaviors in pregnant women with GDM are not clear. The objective of this study was to investigate the pathways by which health literacy, social support, and self-efficacy influence self-management behaviors and the interrelations of the variables, attempt to provide new ideas for improving the self-management behaviors in pregnant women with GDM. The research hypothesis for this study is presented from previous studies: (a) Health literacy, social support, and self-efficacy are factors that influence self-management behaviors among pregnant women with GDM; (b) Health literacy directly influences self-management behaviors and influences self-management behaviors by playing the role of mediator of self-efficacy; (c) Social support directly influences self-management behaviors and influences self-management behaviors though the mediation role of health literacy and self-efficacy; (d) Self-efficacy directly influences self-management behaviors.

## 2. Materials and methods

### 2.1. Participants and data collection procedure

This study was a cross-sectional survey study. All subjects met the following criteria: (i) Initial diagnosis of GDM during pregnancy and met the diagnostic criteria for GDM of the IADPSG 2010 (34); (ii) Age ≥ 18 years; (iii) Those who have an elementary understanding of reading and no communication disabilities; and gestational weeks ≥28 weeks. Pregnant women with GDM who had pre-pregnancy diabetes, multiple pregnancies, and combined severe medical, surgical or obstetric complications were excluded. Using a convenience sampling method, women with GDM who underwent obstetric examination or were hospitalized in the West China Second University Hospital, Sichuan University between December 2021 and June 2022 were selected. The study was approved by the Medical Ethics Committee of the West China Second University Hospital, Sichuan University (No. 2021-219), and verbal informed consent was obtained from each of the participants. All the questionnaires were distributed by our team members after obtaining the consent of the pregnant women, and they were instructed to fill them out. A total of 620 pregnant women with GDM were invited to participate in the study, with 565 agreeing and 55 refusing to participate. Questionnaires with greater than 10% missing items or greater than 50% missing items on any subscale were treated as invalid. Missing values for scales were filled using multiple interpolation method, and missing data for demographic variables are not filled in.

### 2.2. Measures

#### 2.2.1. Demographic characteristics form

Basic demographic information included in age, education level, income level, marital status, occupation, parity, length of pregnancy, family history of diabetes, and so on.

#### 2.2.2. Health literacy scale

This is a multidimensional health literacy scale developed by Japanese scholars Ishikawa et al. (35), based on Nutbean’s Health Literacy Model (36). It consists of 14 items 3 dimensions: functional HL, communicative HL, and critical HL. The Chinese version was translated and culturally adapted by Zhao et al. (37). It’s scored on a four-point Likert scale, with each item rated on a scale of 1–4 from “never” to “often” (functional HL dimension is reverse scored). The final result is typically expressed as the mean score of the 14 items. The Cronbach’s α was 0.853 in our study.

#### 2.2.3. Perceived social support scale

This is a widely used scale to measure social support, developed by Zimet and revised by Zhong et al. (38). The scale has 12 items and is divided into three dimensions: family support, friend support and other support. Each item was rated from 1 to 7 on a 7-point Likert scale from “strongly disagree” to “strongly agree,” with a score range of 7–84. The final result is the sum of the scores of all items, with higher total score indicating stronger social support. The Cronbach’s α was 0.953 in our study.

#### 2.2.4. General self-efficacy scale

General self-efficacy measures, to some extent, the confidence of an individual in the face of a variety of unfamiliar environments or encountering new things, and helps people to develop a comprehensive and in-depth understanding and achieve good results. German scholars Schwarzer and his colleagues developed this scale in 1981, which contains 10 items on a 4-point Likert scale, with each item scoring from 1 to 4 on a scale from “not at all correct” to “completely correct” (39). The Cronbach’s α was 0.930 in our study.

#### 2.2.5. GDM self-management behavior scale

This scale was developed by Li (40), it contains 7 dimensions of diet management, exercise management, weight management, medication use, glucose monitoring, risk assessment and management, and psychosocial adaptation with 37 items to assess the self-management behaviors of GDM patients (The medication use dimension was used to assess the self-management behaviors of pregnant women with GDM who used insulin and other glucose control drugs). The retest reliability coefficient of the total scale was 0.930 and the Cronbach’s α was 0.951. The scale was scored on a 5-point Likert scale, with a total score of 33–185. The level of self-management was determined based on the standardized scores, with scores >80 being good, 60–80 being moderate, and <60 being poor. The Cronbach’s α was 0.938 in our study.

### 2.3. Statistics

SPSS 21.0, AMOS 26.0 ware used for the analysis of the collected data. Descriptive analyses were performed as Mean ± SD. Normality of continuous variables was tested using the kurtosis coefficient method and P–P plot. Continuous variables conforming to a normal distribution were analyzed for correlation using partial correlations, and factors influencing self-management behaviors were analyzed using multiple linear regression. Structural equation modeling was constructed with AMOS 26.0 to perform a path analysis of health literacy, social support, and self-efficacy influencing self-management behaviors in GDM. The maximum likelihood method was chosen as the model parameter estimation method, and the model fitness index was selected and evaluated on the criteria (41): χ^2^/DF < 3.0, RMSEA < 0.08, IFI, TLI, and CFI were > 0.9. *p* < 0.05 indicates statistical significance.

## 3. Results

### 3.1. Demographic characteristics and pregnancy-related conditions

A total of 565 pregnant women with GDM were surveyed in this study, and a total of 523 valid questionnaires were obtained, excluding 42 questionnaires that were not properly or incompletely completed. The average age of the participants was 32.11 ± 3.92 years old, the average length of pregnancy was 35.36 ± 2.91 weeks, 97.9% were Han Chinese, 94.2% lived in urban areas, 98.7% were married, 71.3% had a bachelor’s degree or above, 87.6% were employed, 89.5% had social security, 66.5% had a *per capita* monthly household income >8,000 RMB (equivalent to approximately US$1,160). 68.6% were pregnant with their first child; 93.9% did not use insulin for blood glucose control, and 75.1% had no family history of diabetes.

### 3.2. Descriptive statistics and partial correlations among the variables

Normality test results showed health literacy, social support, self-efficacy, and self-management behavior scores were normally or approximately normally distributed. We then performed a Partial Correlations analysis on the variables, the results are shown in Table 1. After adjusting for variables such as age, health literacy, social support, and self-efficacy were significantly correlated with self-management behaviors of pregnant women with GDM (*r* = 0.533, 0.299, 0.248, respectively; *p* < 0.001), there is also a correlation between each of the two variables (*r* ranging from 0.203 to 0.533, *p* < 0.001).

**Table 1:** The descriptive results and partial correlations among the variables (*n* = 523).

Variables	Mean ± SD	Health literacy	Social support	Self-efficacy	Self-management behavior
Health literacy	3.26 ± 0.41		0.337**	0.203**	0.533**
Social support	65.10 ± 11.25	0.337**		0.373**	0.299**
Self-efficacy	26.64 ± 5.34	0.203**	0.373**		0.248**
Self-management behavior	76.63 ± 12.07	0.533**	0.299**	0.248**	

**p < 0.001; Adjusted variables: age, education level, income level, work status, parity, and family history of diabetes.

### 3.3. Multiple linear regression analysis of the factors influencing self-management behaviors

The self-management behavior score was included as a dependent variable in the multivariate linear regression equation, and the three dimensions of health literacy, social support and self-efficacy scores were included as independent variables. The results showed that there was no multicollinearity among the variables, and all three variables were influential factors in self-management behaviors, with an adjusted *R*^2^ of 0.363, indicating that health literacy, social support, and self-efficacy together accounted for 36.3% of the variance in self-management behaviors. Among these variables, communicative health literacy and critical health literacy explained the strongest self-management behaviors (*β* = 0.316 and 0.255, respectively, *p* < 0.001) (Table 2).

**Table 2:** Multiple linear regression analysis of factors influencing self-management behaviors (*n* = 523).

Variables	*B*	SE	*β*	*t*	*p* value	95.0% CI		Tol	VIF
(Constant term)	17.714	3.722		4.759	<0.001	10.401	25.026		
Functional HL	2.388	0.704	0.121	3.390	0.001	1.004	3.771	0.962	1.039
Communicative HL	6.815	0.927	0.316	7.352	<0.001	4.994	8.636	0.659	1.518
Critical HL	5.130	0.832	0.255	6.168	<0.001	3.496	6.765	0.714	1.401
Self-efficacy	0.216	0.086	0.096	2.501	0.013	0.046	0.386	0.833	1.201
Social Support	0.095	0.043	0.090	2.229	0.026	0.011	0.179	0.745	1.341

*R*^2^ = 0.369, Adjusted *R*^2^ = 0.363, *F* = 90.145, *p* < 0.001; Durbin-Watson = 2.058.

### 3.4. The pathway analysis between health literacy, social support, self-efficacy and self-management behaviors

**Figure 1:**
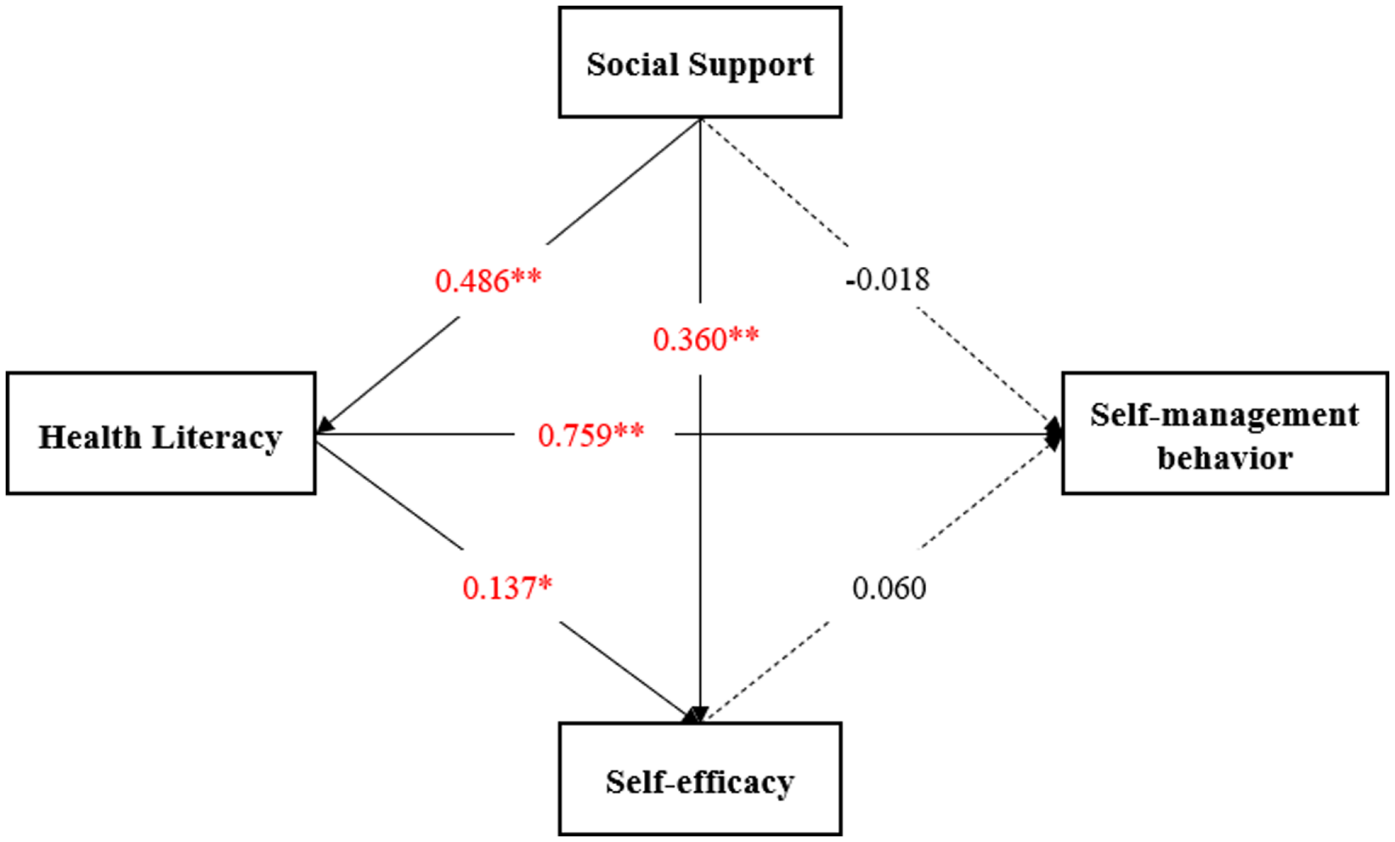
The SEM on the relation between health literacy, social support, self-efficacy and self-management behavior. *p < 0.05, **p < 0.001.

The SEM model was fitted using the maximum likelihood method, and the SEM model was suitable for χ^2^/DF = 2.860, RMSEA = 0.060, IFI = 0.953, TLI = 0.943, and CFI = 0.952. As shown in Figure 1, four paths were statistically significant (*P* < 0.05), health literacy had a direct positive effect on self-management behaviors and self-efficacy (direct effect coefficient = 0.759 and 0.137, respectively, *p* < 0.05), social support had a direct positive effect on health literacy and self-efficacy (direct effect coefficient = 0.486 and 0.360, respectively, *p* < 0.05). Social support and self-efficacy did not have a direct effect on self-management behaviors (*p* > 0.05), but social support could indirectly influence self-management behaviors through the mediating effect of health literacy (Indirect effect coefficient = 0.394) (Table 3). Self-management behaviors of pregnant women with GDM were most influenced by health literacy level, followed by social support. In the pathway of social support affected self-management behaviors, health literacy played a full mediating role, and social support affected self-management behaviors in pregnant women with GDM by influencing their HL.

**Table 3:** The interrelationships among the variables and their effect coefficients (*n* = 523).

Path ends	Effect coefficient	Path beginnings		
		Social support	Health literacy	Self-efficacy
Health literacy	Direct	0.486**	–	–
	Indirect	–	–	–
	Total	0.486	–	–
Self-efficacy	Direct	0.360**	0.137*	–
	Indirect	0.067	–	–
	Total	0.427	0.137	–
Self-management behaviors	Direct	−0.018	0.759**	0.060
	Indirect	0.394	0.008	
	Total	0.377	0.767	0.060

*p < 0.05, **p < 0.001.

## 4. Discussion

In this study, we analyzed the impact of HL, social support, and self-efficacy on self-management behaviors in pregnant women with GDM by constructing an SEM and explored the interplay between the factors, with results that partially supported our research hypothesis: Health literacy and social support are factors affecting self-management behavior in pregnant women with GDM; health literacy directly affects self-management behavior, and social support affects self-management behavior through the mediation of health literacy. It is important to note that HL directly influences GDM self-management behaviors, while social support indirectly influences self-management behaviors through the mediation of HL, which acts as a sufficient mediator between the two. Adequate social support positively affects self-management in pregnant women with GDM through higher levels of health literacy.

Health literacy is an important concept in promoting health behaviors, it can facilitate patient empowerment and adoption of health behaviors that are closely related to people’s health conditions (42). In this study, communicative HL, critical HL, and functional HL were all influential factors in self-management behaviors in pregnant women with GDM, with communicative HL and critical HL having a greater influence on self-management behaviors. Possibly due to the high level of education of the participants in this study. Functional HL primarily assesses basic health-related literacy skills, and our participants are largely free of literacy deficits. Communicative HL and critical HL refer to the dynamic process of obtaining health information from various forms of communication, and critically analyzing the information through rational judgment and applying the processed information to health management, the core of which lies in the effective acquisition, scientific selection and correct application of information (43), these two core health literacies may become more influential on pregnant women with GDM who have a high level of cognitive self-management behavior.

Social support is the perceived and actual instrumental or expressive support provided by communities, social networks, and intimate partners, and social support may indirectly increase maternal psychological well-being by acting as a buffer against the potentially adverse effects of stressful events (44). During pregnancy, pregnant women always require additional psychosocial support to cope with various stressful events related to pregnancy, including support for concerns related to the consequences of illness, interpersonal support, infrastructural support, and health education support (45). Studies have found that social support is strongly associated with pregnancy stress (46), self-care behaviors and self-efficacy during pregnancy (47, 48), affecting maternal quality of life, physical and mental health. Pregnant women with GDM in our study had high levels of social support, but in previous studies, it was found that psychosocial support interventions for pregnant women with GDM tend to focus on information support and there is an underutilization of emotional support such as family (49). enhancing support from spouses or family members could promote self-management, increase psychological resilience, active coping with stress in pregnant women with GDM (50).

The positive impact of HL on self-management behaviors in diabetics has been demonstrated in number of studies. Juul et al. (51) previously investigated 194 Danish patients with type 2 diabetes found that functional HL was associated with following recommended dietary recommendations, functional HL may be an important driver of dietary management behaviors, but no significant association was found between following physical activity recommendations and health literacy (68% with >11 years of education). Souza et al. (52) investigated 129 older adults with type 2 diabetes in Brazil, also found that patients with inadequate functional HL were more likely to have poor glycemic control than those with adequate functional HL, but participants in the study were less educated, with 82.9% having only a high school education or less. Furthermore, health literacy and social support play a joint role in influencing patient self-management behaviors. Zou et al. (53) found that HL and social support in patients with chronic heart failure not only directly influenced the maintenance of self-care, but also indirectly by influencing self-care information. Health literacy and social support were also found to be important factors influencing the decrease in HbA1c levels after hospital discharge for patients with type 2 diabetes (54); they can promote a change in dietary attitudes and seek professional nutrition services among them additionally (55). In contrast to our findings, social support in these studies had a direct effect on patient self-management behaviors and was not mediated through HL. The possible reason for this may be that their participants are mostly older people whose well-managed behavior always needs to be achieved by relying on health care institutions and home environments. In contrast, pregnant women with GDM are younger, have more autonomy in their behaviors and rely more on self-determination to adopt or not to adopt certain health behaviors. HL plays an extremely important role in the health decision-making process (56), thus the precondition for the facilitating effect of favorable social support on self-management behaviors is that pregnant women need to have favorable HL to enable them to make informed self-management decisions.

Our study also confirmed the positive effects of social support and HL on self-efficacy in pregnant women with GDM. HL had a weaker effect on self-efficacy and acted as a partial mediator in the process of social support influencing self-efficacy. Moghadam et al. (57) found that the dimensions of social capital such as community involvement, neighborhood, family and friends, tolerance of diversity, and work relationships were influential factors in the self-efficacy of women with GDM, the enhancement of women’s social capital may increase their self-efficacy in controlling GDM. Self-efficacy represents the confidence and ability of pregnant women with GDM to engage in self-management in some extent, which can alleviate the stress level, improve the quality of life of women with GDM (46), and motivate them to adopt a healthy lifestyle after diagnosis (58). We should focus on the positive impact of well-established social support systems on the self-efficacy of pregnant women with GDM to ensure maternal and infant safety.

Based on our findings, we recommend that health care providers emphasize the active role of HL and social support in actions to improve self-management behaviors in pregnant women with GDM. Improving HL level should be a key point of intervention in practice, and social support systems should be fully mobilized to enhance emotional support and life support to promote improved self-management behaviors.

## 5. Limitations and future directions

There are several limitations to the current study. First, the study recruited participants from a tertiary teaching hospital in Chengdu, Sichuan Province, and this sample only reflects the situation in southwest China. Second, all information was obtained from questionnaires, which were filled out by pregnant women themselves, and some questions may be subject to recall bias. Third, this study is a survey study and causal interpretation may be inadequate. Therefore, we hope that further studies in other types of hospitals and in other regions of China with prospective cohort studies will yield more reliable results.

## Data availability statement

The raw data supporting the conclusions of this article will be made available by the authors, without undue reservation.

## Ethics statement

The studies involving humans were approved by the Medical Ethics Committee of the West China Second University Hospital, Sichuan University/West China Second University Hospital. The studies were conducted in accordance with the local legislation and institutional requirements. Written informed consent for participation was not required from the participants or the participants’ legal guardians/next of kin because there was no potential harm to the patients in this study, and all were fully informed before the survey and verbal consent was obtained from the study participants.

## Author contributions

FT and XZ contributed to conception and design, investigation, data analysis and interpretation, and drafted manuscript. DL and XG contributed to conception and design, data curation, methodology, and critically revised manuscript. SL contributed to investigation and data curation. All authors contributed to the article and approved the submitted version.
